# Sequential vs concurrent adjuvant chemotherapy of anthracycline and taxane for operable breast cancer

**DOI:** 10.1186/s12957-021-02150-4

**Published:** 2021-02-18

**Authors:** Wanjing Chen, Qian Tu, Yanfei Shen, Kejun Tang, Mengying Hong, Yong Shen

**Affiliations:** 1 Department of General Surgery, The Second Hospital of Anhui Medical University, Hefei, 230601 Anhui China; 2 Department of Intensive Care Unit, Zhejiang Hospital, Zhejiang, 322100 Hangzhou China; 3 Department of Surgery, Women’s Hospital, School of Medicine, Zhejiang University, Hangzhou, China; 4 Cancer Institute (Key Laboratory of Cancer Prevention and Intervention, China National Ministry of Education, Key Laboratory of Molecular Biology in Medical Sciences, Zhejiang Province, China), The Second Affiliated Hospital, Zhejiang University School of Medicine, Zhejiang, 310009 Hangzhou China; 5 Department of Breast Surgery, The First Affiliated Hospital, Zhejiang Chinese Medical University, Zhejiang, 310006 Hangzhou China

**Keywords:** Breast cancer, Sequential regimen, Concurrent regimen, Anthracycline, Taxanes

## Abstract

**Background:**

Whether a sequential or concurrent regimen of anthracyclines and taxanes is superior for breast cancer is controversial. We compared the efficacy of two regimens in patients with operable breast cancer based on all relevant published data of phase III randomized controlled trials.

**Methods:**

A comprehensive literature search on PubMed, Web of Science, Embase, ScienceDirect, Google Scholar, and ClinicalTrials.gov databases was performed up to May 2020. Meta-analysis was performed to evaluate the different efficacy on disease-free survival (DFS) and overall survival (OS) for the two chemotherapy regimens. Subgroup analyses were further carried out in terms of node status and anthracycline selection.

**Results:**

Compared to the concurrent regimen, the sequential regimen did not improve the DFS or OS in the population studied. Subgroup analysis showed that in node-positive patients, the sequential regimen had better DFS, but not OS, than the concurrent regimen. In sequential regimen, patients who received doxorubicin and taxanes had improved DFS and OS than patients who were administered epirubicin and taxanes. Furthermore, for patients who received doxorubicin and taxanes, compared to the sequential regimen, fewer cycles (4 cycles) of concurrent treatment resulted in a worse DFS and OS, which can be rescued by more cycles (6 cycles).

**Conclusions:**

The sequential regimen of anthracyclines and taxanes for patients with operable breast cancer did not yield a significant benefit in DFS or OS over the concurrent regimen. The sequential regimen, however, provided a better DFS than concurrent regimen for node-positive patients. Interestingly, further subgroup analysis showed that for node-positive patients who were given doxorubicin and taxanes, more cycles (6 cycles) of the concurrent regimen may rescue the efficacy for fewer cycles (4 cycles).

## Introduction

Breast cancer is the most common cancer in women worldwide. In 2018, 266,120 new breast cancer cases occurred in the USA, accounting for 30% of all female malignant tumors, and 40,920 deaths, accounting for 14% of the total mortality of female malignancies [1]. In China, the incidence and the mortality of female breast cancer is 41.82/100,000 and 9.91/100,000, respectively [2]. Despite the great advances achieved in diagnosis and treatment, breast cancer remains one of the leading causes of cancer-related deaths [1]. A number of studies have indicated that adjuvant chemotherapy benefits for early breast cancer patients after surgery [3]. Currently, anthracyclines and taxanes are the basic components in chemotherapy because the addition of a taxane to an anthracycline-containing regimen is associated with better RFS and OS [4–7]. Although the regimens containing an anthracycline and taxane have been reported to be more effective, the optimal schedule of drug intervention (sequential or concurrent) remains questionable. For example, a concurrent regimen requires lower dose of drug, which may affect the efficiency. Nevertheless, the sequential administration may provide an optimal dose for each compound, but requires a longer time duration of treatment.

Thus, to elucidate which regimen offers a greater benefit for patients, we performed this meta-analysis to comprehensively evaluate the clinical effect of these two adjuvant regimens in patients after breast cancer surgery by including all relevant phase III randomized control studies.

## Methods

The methods used for this meta-analysis and generation of inclusion criteria were based on PRISMA (Preferred Reporting Items for Systematic Reviews and Meta-Analyses) recommendations.

### Literature search strategy

Databases including PubMed, Web of Science, Embase, ScienceDirect, Google Scholar, and ClinicalTrials.gov up to May 2020 were the basis for the literature search, with the following keywords: “breast cancer,” “sequential and (concurrent or concomitant),” “adjuvant chemotherapy,” “anthracyclines and taxanes,” and “(doxorubicin or epirubicin) and (docetaxel or paclitaxel).” In addition, the references of relevant reviews were searched for additional studies.

### Inclusion and exclusion criteria [8]

The inclusion criteria were as follows: (1) phase III randomized control studies; (2) breast cancer that had not spread beyond the breast or the axillary lymph nodes; and (3) patients who underwent curative surgical resection and were subsequently randomized to receive the sequential or concurrent regimen. Standard post-operative radiotherapy and endotherapy protocols, with tamoxifen or aromatase inhibitors, were permitted.

The exclusion criteria were as follows: (1) abstract only; (2) duplicate publications; (3) reviews, letters, or comments; and (4) no available data.

### Data extraction

Two investigators independently screened all the studies and extracted data. Differences were resolved by discussion until obtaining consensus. The following data were extracted and recorded in a predesigned form: study design, year of reporting, regimen details, median follow-up, hazard ratio (HR) of DFS and OS, and the number of outcome events.

### Quality assessment

We used The Cochrane Collaboration “Risk of bias” assessment tool to assess the potential sources of bias in the included studies [7]. Two authors independently assessed the potential risk of bias for each study; any differences in judgment were resolved through discussion. The domains were assessed according to random sequence generation, allocation concealment, blinding of participants and personnel, blinding of outcome assessment, incomplete outcome data, selective reporting, and other bias. We assigned ratings of “high,” “low,” or “unclear” risk of bias to each domain for the included studies.

### Statistical analysis

RevMan 5.3 was used for performing this meta-analysis. The *I*^2^ and Cochrane *Q* tests were used to assess heterogeneity among the included studies, with a *P* < 0.1 or *I*^2^ > 50% considered to be significant. The risk ratio (RR), as well as the corresponding 95% confidence intervals (CIs), was pooled by an appropriate model (fixed- or random-effects model) based on the results of the heterogeneity test. A *Z* test was used to evaluate the significance of the pooled effect size. For dichotomous variables, a Mantel–Haenszel rate ratio with 95% CIs was calculated. If significant heterogeneity was detected (*P* < 0.1), causes of heterogeneity were subsequently determined via subgroup analyses; otherwise, a random effect model was selected. For continuous variables, we used a fixed effect weighted mean difference (WMD) for measurements and the 95% CIs were calculated.

All analyses were performed according to the intention-to-treat principle when appropriate data were available. The publication bias was evaluated by the Egger’s and Begg’s tests using the Stata 11.0 software. The sensitivity analyses were performed by omitting each individual study at a time. For these analyses, a *P* < 0.05 indicated statistical significance.

## Results

### Characteristics of the included studies

**Fig. 1:**
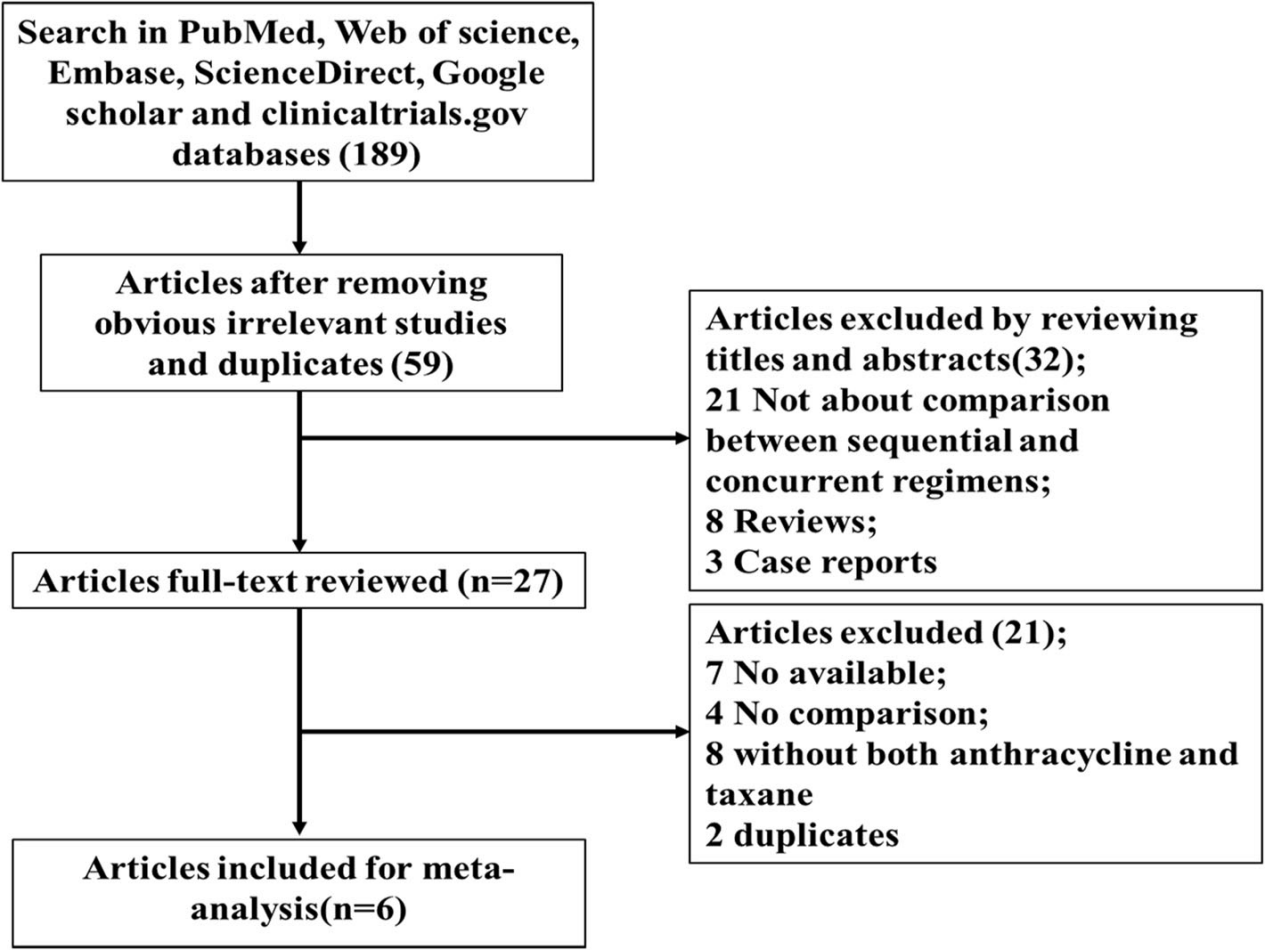
Flow diagram of the study selection process

After an initial literature search on PubMed, Web of Science, Embase, ScienceDirect, Google Scholar, and ClinicalTrials.gov databases, 189 articles were identified. After excluding duplicates and irrelevant studies, 59 potentially relevant articles remained. Of the 59 articles, 32 were further excluded due to mis-matching contents (21 studies did not report the comparison between sequential and concurrent regimens; 8 reviews and 3 case reports). For the remaining 27 articles, another 21 studies were excluded for the following reasons: no available data (*n* = 7); no comparison between sequential and concurrent regimens (*n* = 4); regimens did not contain an anthracycline and taxane (*n* = 8); and duplicates (*n* = 2). Finally, 6 articles were included in this meta-analysis [9–14] (Fig. 1).

Among the 6 studies, a total of 6866 breast cancer patients after surgery were given the sequential regimen of an anthracycline and taxane as adjuvant chemotherapy, while 6847 patients received concurrent treatment (Table 1). The publication years ranged from 2010 to 2017. All of the studies were phase III randomized control trials.

**Table 1:**
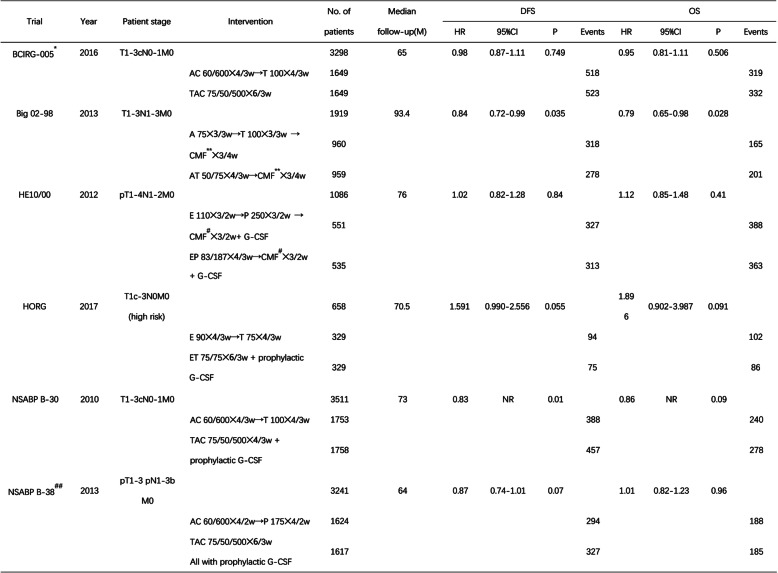
Main characteristics of the studies included in this meta-analysis

BCIRG-005* either arm with G-CSF at the discretion of the investigator; CMF** patients in all arms received three cycles of CMF that were given every 4 weeks as oral cyclophosphamide at 100 mg/m^2^ on days 1–14 and intravenous methotrexate at 40 mg/m^2^ plus intravenous 5-fluorouracil at 600 mg/m^2^ on day 1 and day 8; *CMF*^*#*^ intensified CMF (cyclophosphamide at 840 mg/m^2^, methotrexate at 57 mg/m^2^, and 5-fluorouracil at 840 mg/m^2^); *NSABP B-38*^*##*^ all patients receive primary prophylaxis with pegfilgrastim or filgrastim; *NR* no report; *A* doxorubicin; *E* epirubicin; *C* cyclophosphamide; *T* docetaxel; *P* paclitaxel

### Quality assessment

**Fig. 2:**
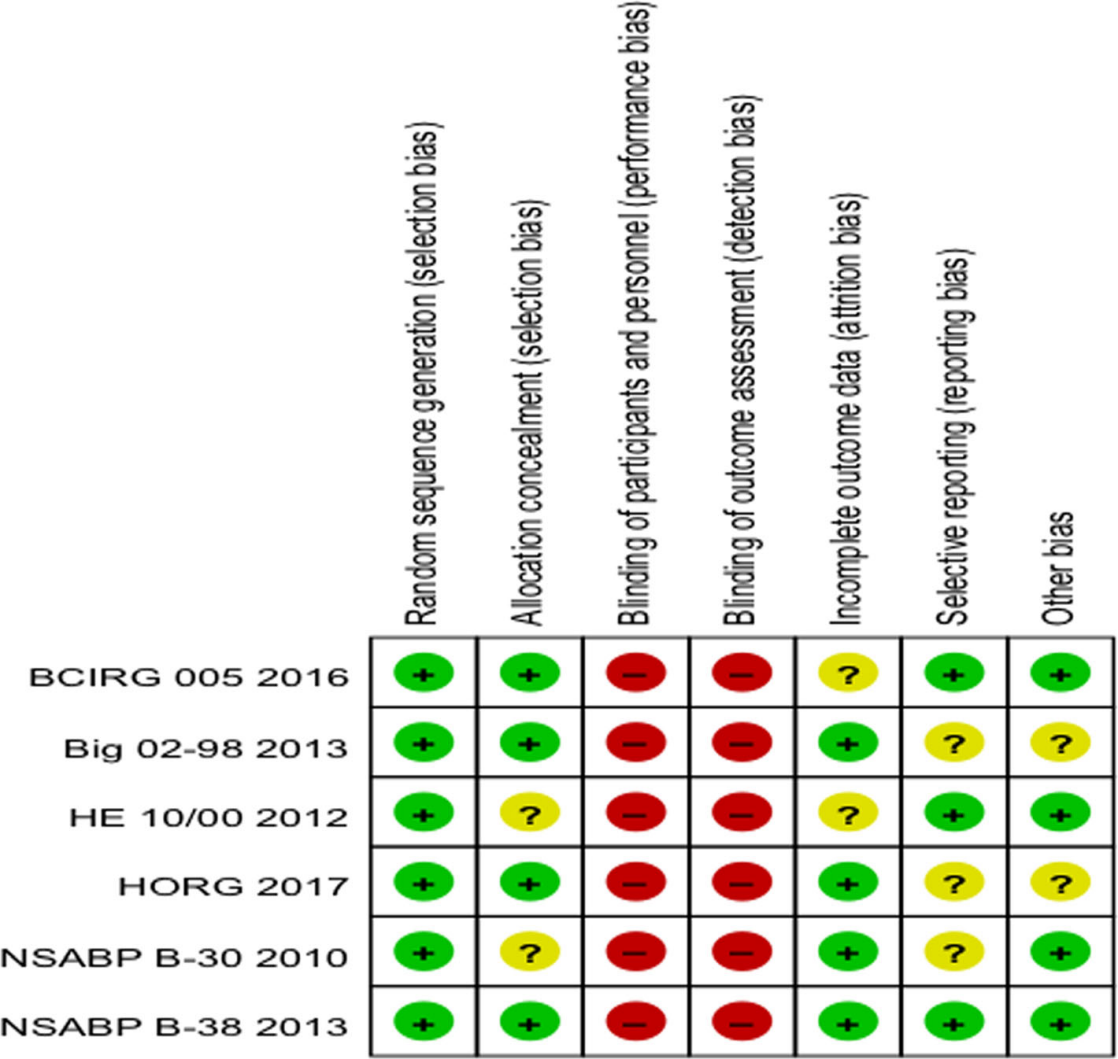
“Risk of bias” assessment for each risk of bias item of each included study

The detail of the risk of bias summary is outlined in (Fig. 2). All studies were considered to have a median risk of bias. Randomized sequence generation was implemented in all 6 studies, and 4 studies implemented allocation concealment. All studies were conducted on the intention-to-treat principle. None of the 6 studies were blinded to the participants or the outcome assessment.

### Meta-analysis for DFS

**Fig. 3:**
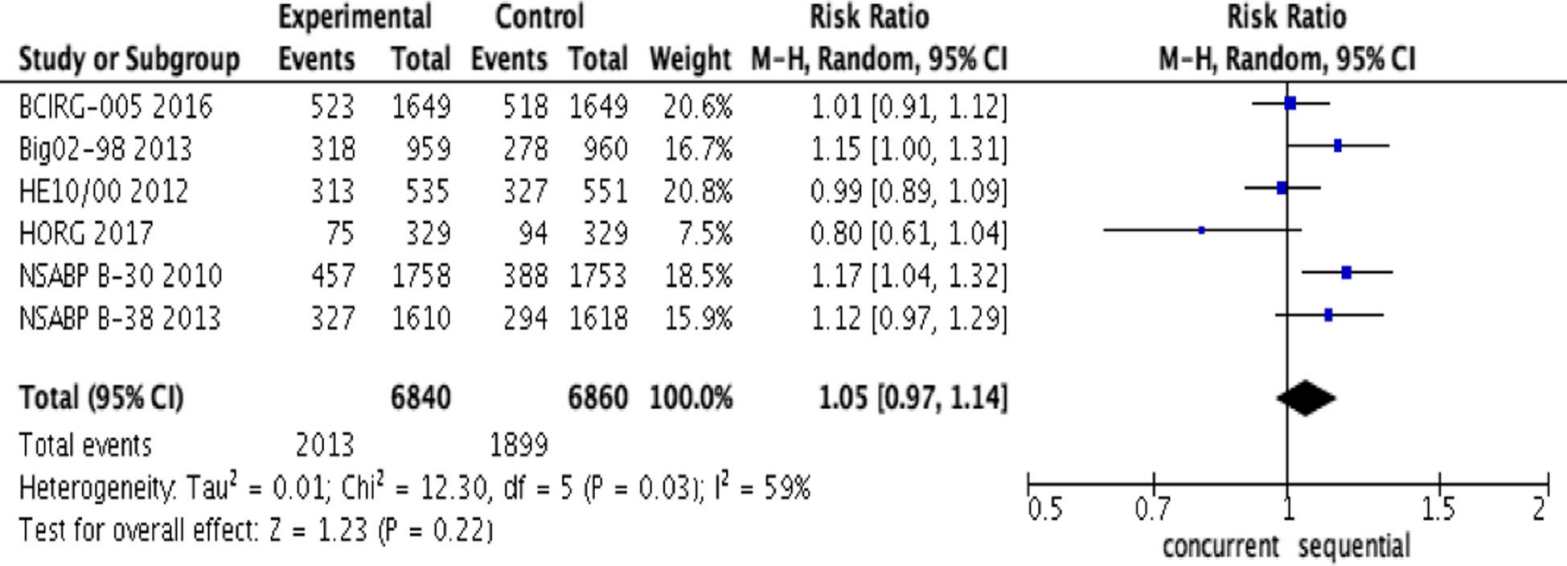
Forest plots of the pooled RR for disease-free survival (DFS) of concurrent regimens and sequential regimens. The results indicated that there was no significant difference in DFS between concurrent and sequential groups (RR, 1.05; 95% CI, 0.97-1.14; *P* = 0.22)

Significant heterogeneity among studies (*I*^2^ = 59%, *P* = 0.03; Fig. 3) was demonstrated in the analysis of DFS between the sequential and concurrent regimens; thus, we used the randomized effects model to pool the RR. The meta-analysis showed that sequential regimens of anthracycline and taxane appeared not to add significant improvement in DFS over the concurrent regimen (RR, 1.05; 95% CI, 0.97-1.14; *P* = 0.22, Fig. 3).

### Meta-analysis for OS

**Fig. 4:**
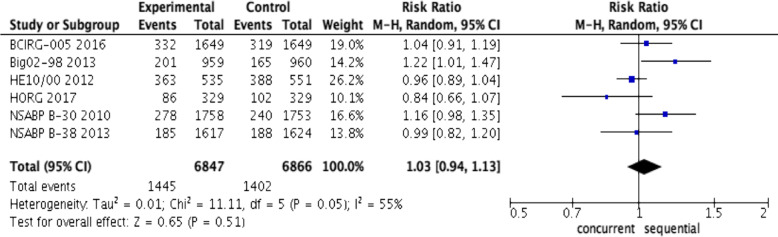
Forest plots of the pooled RR for overall survival (OS) of concurrent regimens and sequential regimens. The results indicated that there was no significant difference in OS between concurrent and sequential groups (RR, 1.03; 95% CI, 0.94 to 1.13, *P* = 0.51)

Significant heterogeneity (*I*^2^ = 55%, *P* = 0.05, Fig. 4) was observed among studies for OS in comparison between the sequential and concurrent regimens; thus, the randomized effects model was used. The pooled estimate showed that there was no significantly improved OS between sequential regimens and concurrent regimens (RR, 1.03; 95% CI, 0.94 to 1.13, *P* = 0.51, Fig. 4).

### Sub-analysis in node status for DFS and OS

**Fig. 5:**
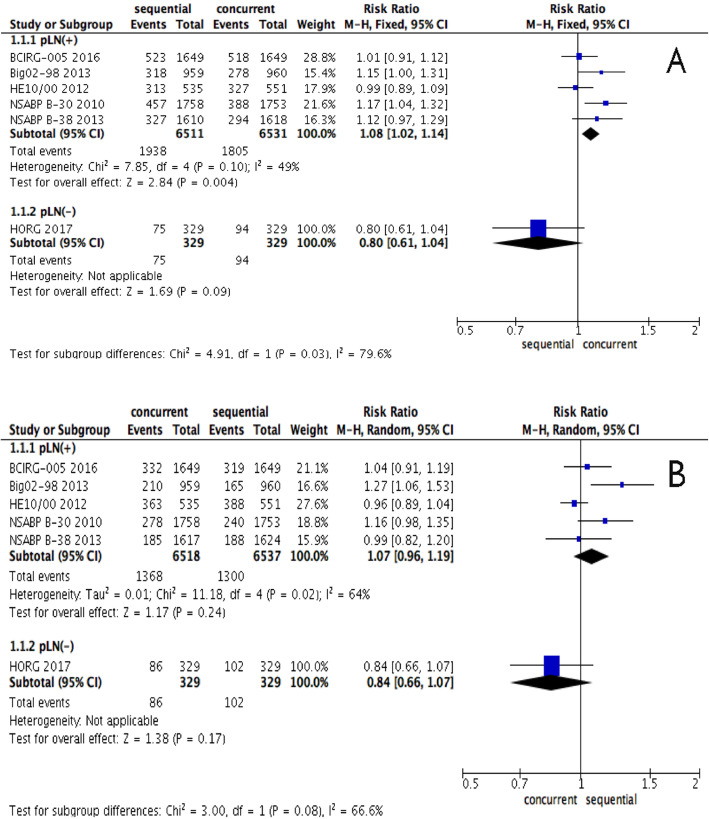
**a** Forest plots of the pooled RR for the sub-analysis of node status effect in DFS between sequential and concurrent regimens. The results indicated that there was a significantly better DFS in patients with node-positive status who were administrated a sequential regimen (RR, 1.08; 95% CI, 1.02-1.14, *P* = 0.004). **b** Forest plots of the pooled RR for the sub-analysis of node status effect in DFS between sequential and concurrent regimens. The results indicated that there was no significant difference in OS in patients with node-positive/negative status between concurrent and sequential regimens (RR, 1.07; 95% CI, 0.96-1.19, *P* = 0.24)

The eligible patients in the HORG trial [12] were early breast cancer patients at high risk and axillary lymph node-negative status, while the other trials included patients with node-positive status. We conducted a sub-analysis according to the axillary lymph node status. The pooled estimate showed that there was a significantly better DFS in patients with node-positive status who were administrated a sequential regimen (RR, 1.08; 95% CI, 1.02-1.14, *P* = 0.004, Fig. 5a), yet the OS was similar for both regimens (RR, 1.07; 95% CI, 0.96-1.19, *P* = 0.24, Fig. 5b).

**Fig. 6:**
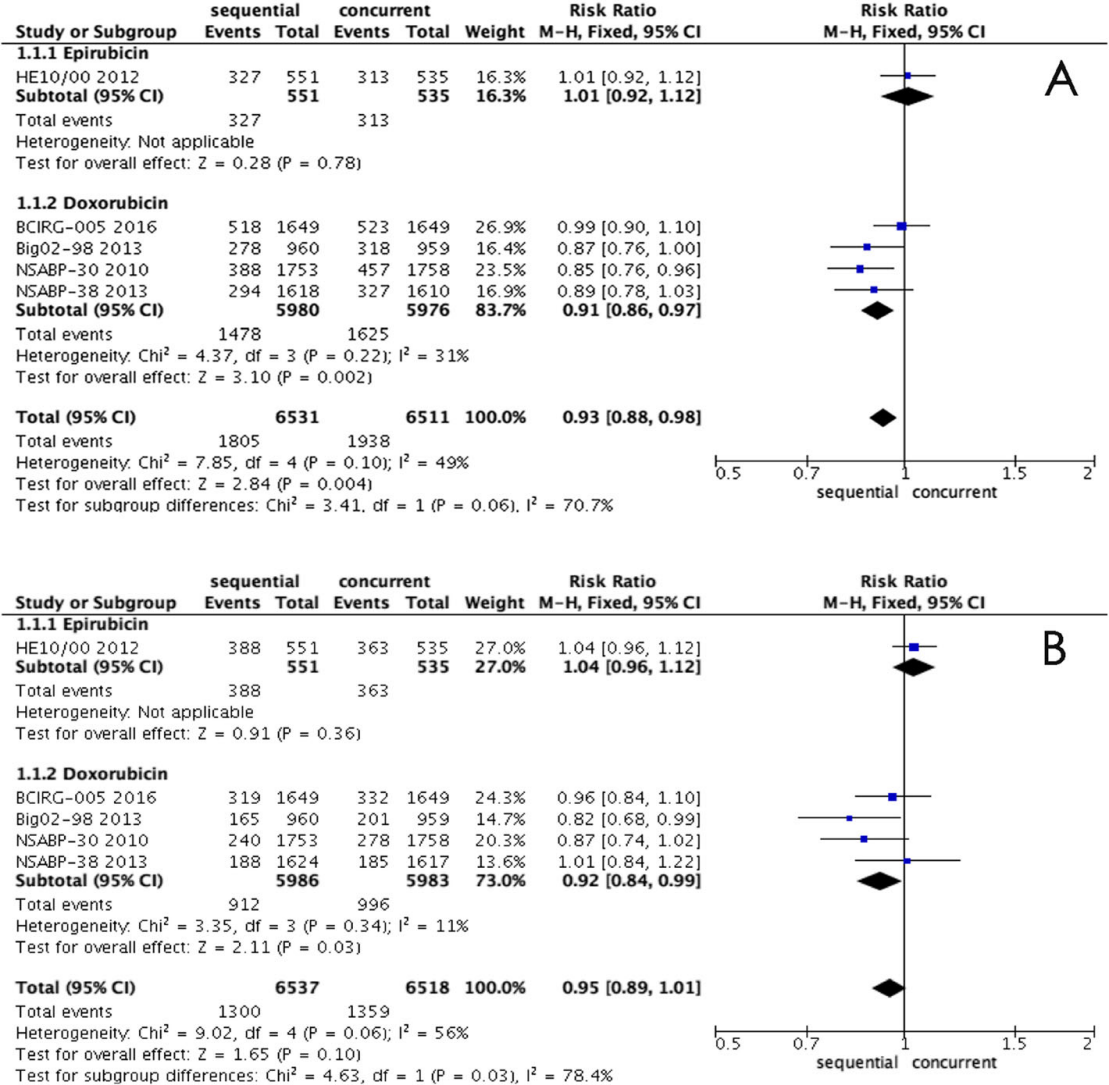
**a** Forest plots of the pooled RR for the sub-analysis of anthracycline effect in DFS between sequential and concurrent regimens. The results indicated that there was a significantly better DFS in patients with doxorubicin and a taxane in the sequential group (RR, 0.91; 95% CI, 0.86-0.97, *P* = 0.002). **b** Forest plots of the pooled RR for the sub-analysis of anthracycline effect in OS between sequential and concurrent regimens. The results indicated that there was a significantly better OS in patients with doxorubicin and a taxane in the sequential group (RR, 0.92; 95% CI, 0.84-0.99, *P* = 0.03)

The choice of anthracyclines may be another reason causing heterogeneity. Epirubicin was selected in the HE10/00 trial [14], while the other four trials used doxorubicin among patients with node-positive status. The sub-analysis data according to the choice of anthracycline showed that there was no significant heterogeneity; however, a better DFS (RR, 0.91; 95% CI, 0.86-0.97, *P* = 0.002, Fig. 6a) and OS (RR, 0.92; 95% CI, 0.84-0.99, *P* = 0.03, Fig. 6b) were achieved in patients with doxorubicin and a taxane in the sequential group.

**Fig. 7:**
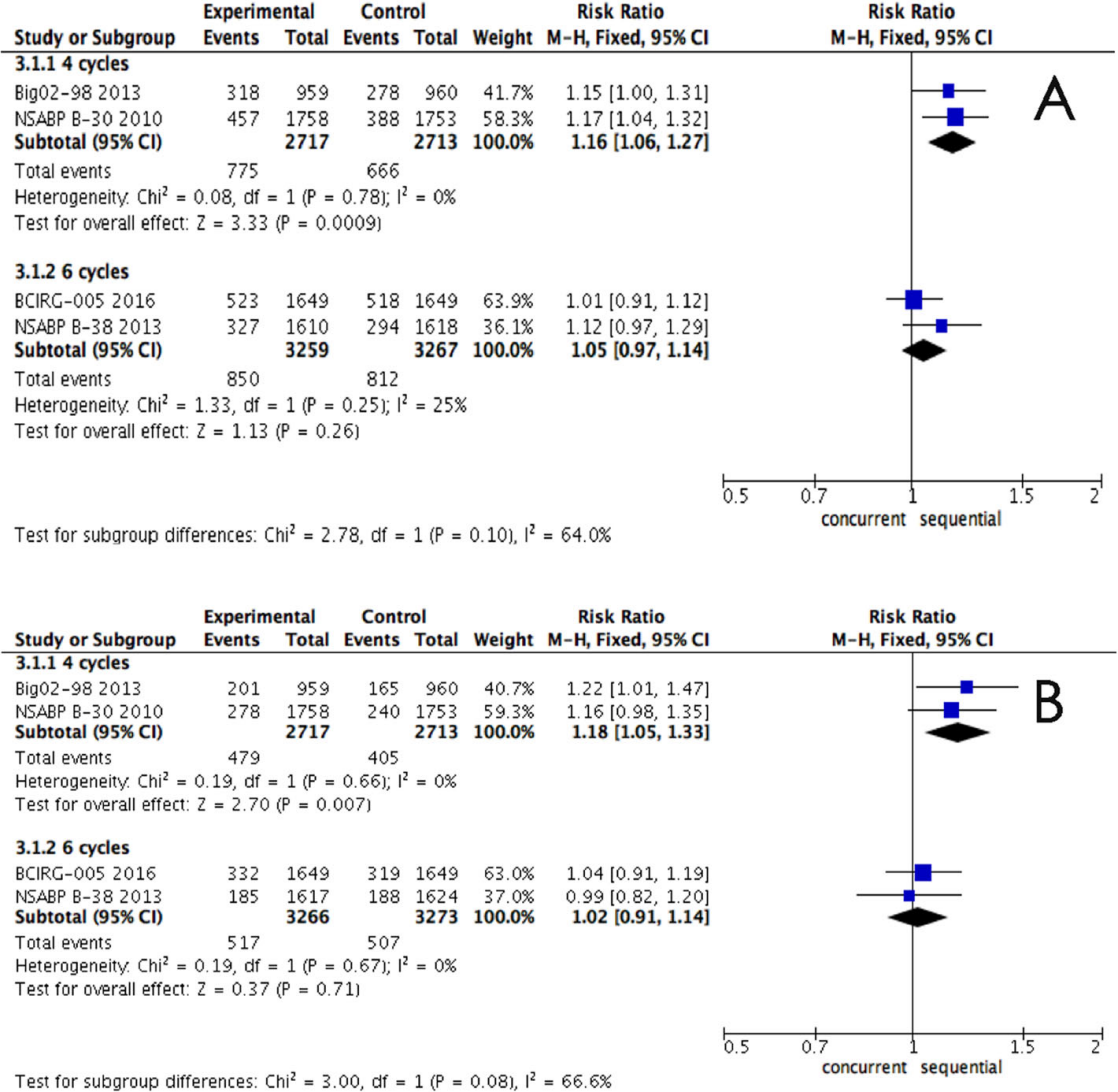
**a** Forest plots of the pooled RR for the sub-analysis of cycle number effect in DFS in epirubicin arms between sequential and concurrent regimens. The results indicated that fewer cycles (4 cycles) of concurrent treatment had worse DFS (RR, 1.16; 95% CI, 1.06-1.27, *P* = 0.0009). **b** Forest plots of the pooled RR for the sub-analysis of cycle number effect in DFS in epirubicin arms between sequential and concurrent regimens. The results indicated that fewer cycles (4 cycles) of concurrent treatment had worse OS (RR, 1.18; 95% CI, 1.05-1.33, *P* = 0.007)

The cycles of concurrent regimen with doxorubicin and a taxane also appeared to affect heterogeneity. The patients in the Big02-98 [11] and NSABP B-30 trials [10] received 4 cycles of doxorubicin and taxanes, while patients in the other two trials [9, 13] were treated for six cycles. Therefore, we then conducted another sub-analysis. The pooled estimate showed that fewer cycles (4 cycles) of concurrent treatment had worse DFS (RR, 1.16; 95% CI, 1.06-1.27, *P* = 0.0009, Fig. 7a) and OS (RR, 1.18; 95% CI, 1.05-1.33, *P* = 0.007, Fig. 7b) compared to sequential regimen, whereas more cycles (6 cycles) rescued the loss.

### Sensitivity analysis and publication bias

**Fig. 8:**
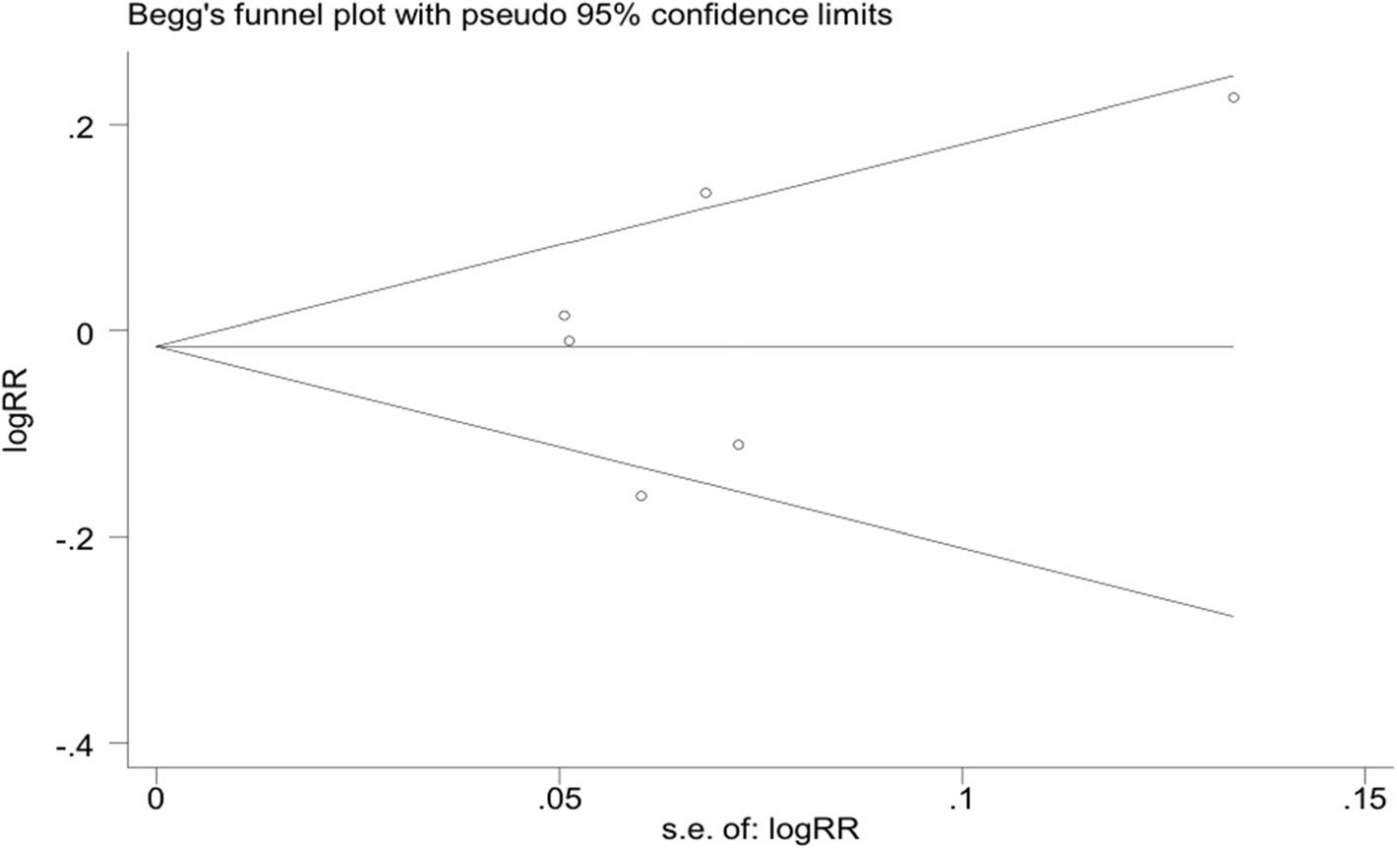
Funnel plot based on the risk ratio (RR) of disease-free survival (DFS) showing no publication bias among the included studies

Sensitivity analysis showed that there was no significantly different incidence through omitting each study. No significant publication bias was found based on the Egger’s and Begg’s test (*P* > 0.05, Fig. 8).

## Discussion

Whether sequential or concurrent usage of anthracyclines and taxanes contributes more for operable breast cancer patients’ survival is controversial. Our meta-analysis presented evidences that the sequential regimen is not associated with a superior DFS or OS than the concurrent regimen from any cause, according to all published data of phase III randomized controlled trials.

Considering the importance of axillary lymph node status on breast cancer recurrence, DFS, and OS, we conducted a sub-analysis to illuminate whether node-positive or node-negative would affect the result. Data from 5 included phase III trials showed that in node-positive patients, sequential treatment provided a statistically better DFS. We further conducted another subgroup analysis in node-positive patients with respect to different choices of an anthracycline. In particular, data from 4 trials [9–11, 13] showed that patients treated with doxorubicin had a better DFS and OS than patients who were treated with epirubicin. Interestingly, in the doxorubicin group given four cycles of drug treatment [10, 11], patients in the sequential arm achieved better DFS and OS compared to the combination arm, whereas patients receiving six concurrent cycles had a similar survival rate as the sequential group [9, 13]. This finding may be explained in two ways. First and most importantly, cumulative doses of drugs were the main factor. For patients in Big 02-98 [11] and NSABP B-30trials [10], the sequential arms were delivered with higher cumulative doses of both doxorubicin (225 vs 200 mg/m^2^ in Big02-98 trial, 240 vs 200 mg/m^2^ in NSABP B-30 trial) and docetaxel (300 vs 300 mg/m^2^ in Big02-98 trial, 400 vs 300 mg/m^2^ in NSABP B-30 trial). This is consistent with other reports that “lower doses” (30 mg/m^2^) of doxorubicin are correlated with inferior survival compared with “higher doses” (60 and 40 mg/m^2^) [15, 16]. Second, the dose intensity was higher in both doxorubicin (25 vs 16.7 mg/m^2^ per week in Big02-98 trial, 20 vs 16.7 mg/m^2^ per week in NSABP B-30 trial) and docetaxel (33.3 vs 25 mg/m^2^ per week in Big02-98 trial and NSABP B-30 trial) in sequential arm, which validated the finding from NEAT (National Epirubicin Adjuvant Trial) trial [17] that a higher dose intensity confers a greater favorable long-term outcome. The principle behind dose density relates to the Gompertzian model and Norton-Simon hypothesis that smaller tumor grows faster so that the regrowth rate is higher between treatment cycles [18, 19], and as tumor shrinks, the regrowth rate increases to make the chemotherapy level capable of initiating regression be insufficient to maintain this regression and produce cure, indicating the regression rate may be overcome by switching to alternative cytotoxic therapy [20]. In contrast, the remaining four trials [9, 12–14] did not show significantly better survival with the sequential regimens than concurrent treatment. Given the patients assigned to the concurrent treatment were administered a higher cumulative dose than the Big02-98 [11] and NSABP-30 trials [10], it may be inferred that once the threshold of total dose is surpassed, higher cumulative doses did not add to efficacy.

According to the different choices of anthracyclines in node-positive patients, we conducted a further sub-analysis that showed patients who received doxorubicin, but not epirubicin, had a better DFS and OS with sequential treatment. This finding may be related to pharmacodynamics and pharmacokinetics [21].

Some disadvantages of this meta-analysis should be noted. First, only a small number of studies were included. Second, the difference in the regimens as opposed to the current regimens which are DD AC followed by weekly T with or without carbo in this sequence or reverse which has been widely adopted by the oncology community may impact the conclusion. Third, heterogeneity, which may affect the results, existed in several trials. The HE 10/00 trial [14] included patients with a pathological stage T4, while the HORG trial [12] focused on patients with early breast cancer as well as node-negative status and high risk. In addition, the choice of anthracyclines and the cycles of treatment in the 6 trials were different. Fourth, subgroup analyses of some confounding factors, such as country, ethnicity, hormonal receptor status, and HER-2 status, could not be performed to determine the influence of these factors due to insufficient data.

In conclusion, breast cancer patients with node-positive status and patients who were given doxorubicin, especially for those desiring fewer cycles of chemotherapy, should be recommended with the sequential regimen. Alternatively, sufficient cycles of concomitant regimen may acquire a similar benefit as the sequential regimen. Concurrent treatment should be administered with G-CSF prophylactically. Additional RCTs with larger sample sizes should be performed to verify the results of this meta-analysis.

## Data Availability

The datasets used and analyzed during the current study are available from PubMed, Web of Science, Embase, ScienceDirect, Google Scholar, and ClinicalTrials.gov.

## Abbreviations

DFS: Disease-free survival
OS: Overall survival
HR: Hazard ratio
RR: Risk ratio
CIs: Confidence intervals
WMD: Weighted mean difference
